# Patterns and underlying mechanisms of non-volant small mammal richness along two contrasting mountain slopes in southwestern China

**DOI:** 10.1038/s41598-017-13637-0

**Published:** 2017-10-16

**Authors:** Zhongzheng Chen, Kai He, Feng Cheng, Laxman Khanal, Xuelong Jiang

**Affiliations:** 1 State Key Laboratory of Genetic Resources and Evolution, Kunming Institute of Zoology, Chinese Academy of Sciences, Kunming, Yunnan 650223 China; 2 Institute of Health Sciences, Anhui University, Hefei, Anhui 230601 China; 3 Central Department of Zoology, Tribhuvan University, Kathmandu, 44613 Nepal

## Abstract

The species richness patterns of small mammals and the processes shaping them in two gradients of a mountain with different spatial and climatic characteristics were examined using standard sampling scheme. We trapped 2,006 small mammals representing 37 species, along elevational gradients on both western and eastern slopes of the Ailao Mountains, Southwest China. Using mid-domain effect model, model selection and model averaging, we examined the effects of slope, area, mean annual temperature (MAT), mean annual humidity (MAH), productivity, plant species richness (PSR) and the mid-domain effect (MDE) on the patterns of small mammal diversity. The hump-shaped patterns were favored along the elevational gradient, but shapes of diversity curves were different on the contrasting slopes. Area and productivity were the most important factors in explaining the variation of total species richness. However, for each specific group of small mammals (i.e. insectivores vs. rodents, large-ranged vs. small-ranged species, endemic vs. non-endemic species), the peaks of species richness and their primary drivers varied. The major explanatory factors for richness pattern of each small mammal group were not significantly different between the slopes, suggesting the existence of the general underlying mechanisms on two slopes of a mountain.

## Introduction

Understanding the patterns of biodiversity variation along environmental gradients is a fundamental question in ecology and biogeography^1,2^. Montane systems, with striking ecological variations along altitudinal belts, are often recognized as biodiversity hotspots that provide an ideal place to document patterns and explore mechanisms for diversity along gradients^3–5^. Rahbek^6,7^classified elevational species richness patterns into four basic types: decreasing, plateau at low elevations then decreasing, hump-shaped, and increasing; the hump-shaped pattern being the most common (*c*. 50%). Different methodological approaches on non-volant small mammals (hereafter- small mammals) have robustly demonstrated the predominance of the hump-shaped pattern^8,9^, with a few exceptions^10,11^. Despite years of research, the mechanism(s) driving species richness patterns along elevational gradients remain poorly understood and controversial^12–14^.

The most frequently tested drivers for diversity patterns are area, the mid-domain effect (MDE), climate, and productivity^4,15–18^. The species-area relationship (SAR) is based on the assumption that larger areas should contain more species^1^, and many studies have demonstrated the importance of the area in shaping elevational species richness patterns^18,19^. The MDE predicts a peak or plateau of species richness in the middle of the domain without any other contributing factors^20^ and has been well-discussed in the last decade^8,21,22^. Empirical support for MDE has been demonstrated in many studies along elevational gradients^21,23^, although there are many controversies about its predictions^22^. However, as a null model, MDE should not be simply rejected or accepted^15^.

Climatic factors (e.g. temperature, rainfall, and humidity) can have strong effects on species richness, influencing it both directly (e.g. the physiological limitations of temperature) and indirectly (e.g. resources responding to climate)^2^. However, because of the lack of small-scale climatic data, it is difficult to quantify these climatic hypotheses and to disentangle the relative importance of each climatic factor in shaping diversity along elevational gradients^12,14^. Data from global databases interpolated from weather stations are too coarse-grained for analyzing highly responsive elevational gradients at small spatial scales^8^. Until small-scale climatic variables are obtained, it is not possible to examine the precise roles of these factors^4^. Apparently, only few studies have used field measurements of climatic factors to study elevational richness patterns^3,24^. Productivity has also been frequently cited as the main driver of species richness patterns^11,25^. Productivity hypotheses predict a positive relationship between productivity and abundance; in turn, as abundance increases, so does species richness. Additionally, plant species richness (PSR) has been considered an important factor for the species richness of small mammals^17^. Similar to the climatic factors, lack of robust productivity and vegetation data is a constraint for a quantitative test of its effects on species richness along elevational gradients. Thus, higher quality data including climate, productivity and vegetation are pressingly needed.

Patterns of diversity and their underlying mechanisms may vary among taxa, biogeographical regions and species composition^14,23,26^, and are strongly impacted by sampling scale and technique/effort^7^. Species with large ranges are more likely to produce a hump-shaped richness pattern and fit MDE better than species with smaller ranges^27^. Endemic species richness is expected to have a higher elevational peak and may be more affected by MDE than non-endemics^23^. Dissimilar patterns and sources are also reported when total species of the area are divided into different taxonomic groups^26^. Rahbek^7^ found that diversity patterns and mechanisms are quite different depending on the choice of scale and that uneven sampling can directly influence the results.

**Figure 1:**
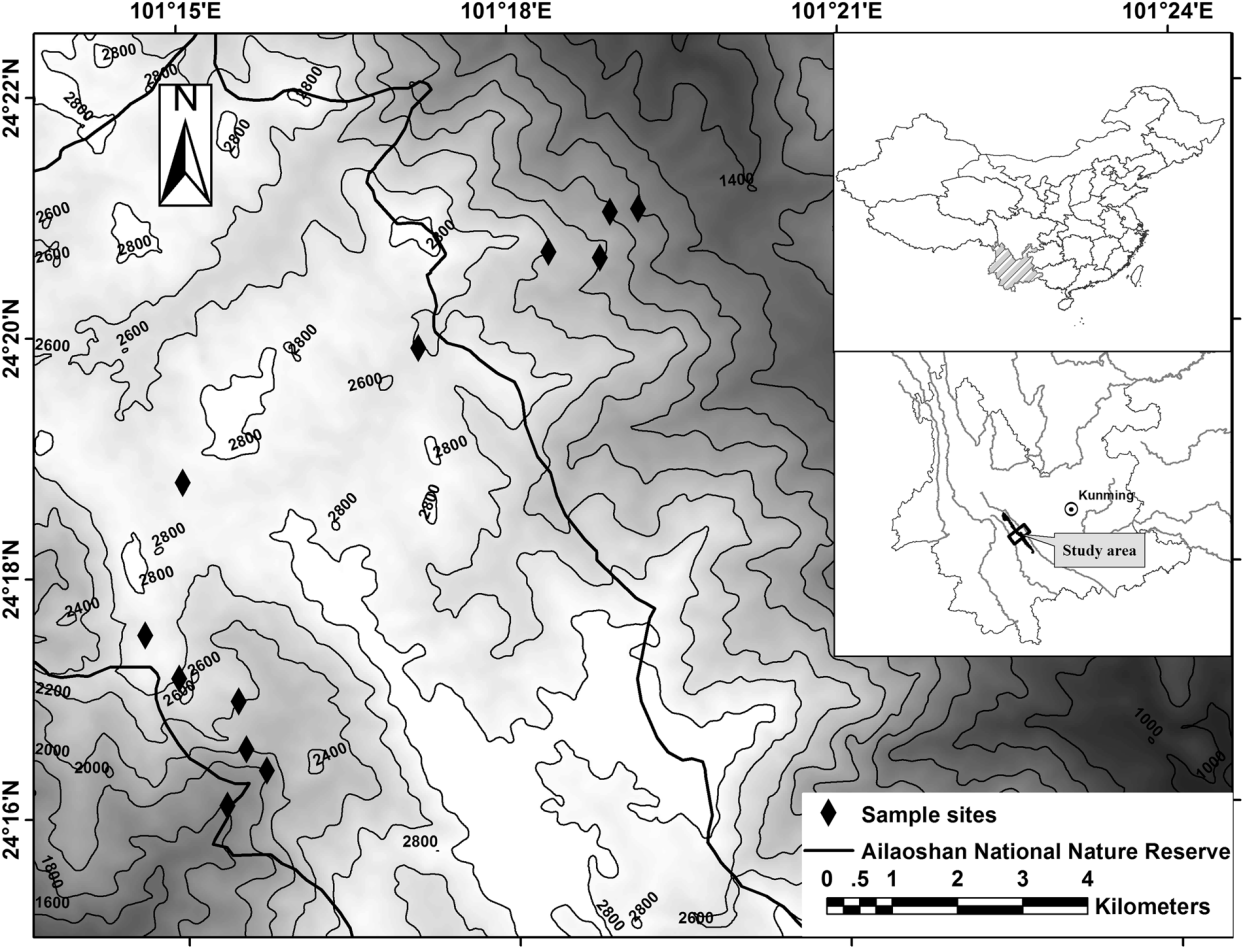
Map of the study area in the Ailao Mountains, Yunnan, China created by ArcGIS 10.3 (ESRI, Redlands, California, USA).

Comparative field studies between different transects/taxa within the same region or among multiple montane regions are essential to further understanding of diversity patterns along elevational gradients^2,5,28^. Owing to its difficulty, however, such studies are remarkably scarce, especially in the southwest China. In the present study, we conducted a comparative analysis of small mammal species richness patterns on both the western and eastern slopes of the Ailao Mountains in southwestern China (Fig. 1). We trapped small mammals using standardized techniques, and recorded detailed temperature and humidity data from the sampling sites. We divided the small mammal species into groups based on their taxonomy, range size, and endemism and analyzed the elevational species richness patterns and their causes. Our aims were to: 1) explore the small mammal richness patterns and causes along the elevational gradients of two opposite faces of the Ailao Mountains, 2) assess if the small mammal richness patterns and underlying mechanisms along elevational gradients were common between the two contrasting slopes within the same mountain range but different spatial and climatic conditions?

## Results

### Small mammal fauna and species richness pattern

**Figure 2:**
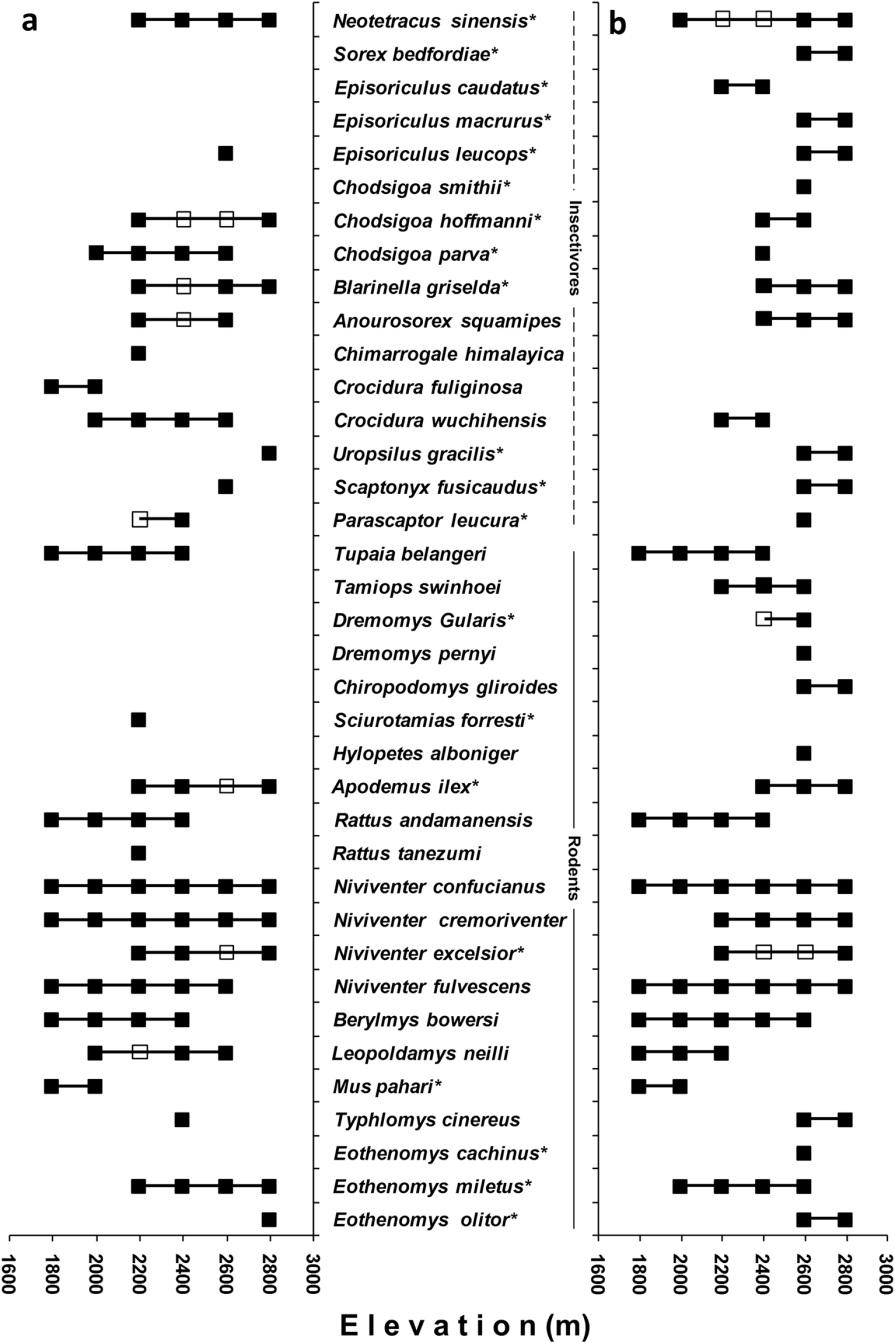
Elevational distribution range, taxonomy and endemism of each small mammal in the Ailao Mountains for (**a**) western slope and (**b**) eastern slope. Solid squares indicate sites the elevation at which individuals were trapped or sighted, and hollow squares indicate an interpolated site based on the presence of the species at both lower and upper localities. “*” indicates the endemic species.

In total, we trapped 2,006 individuals representing 37 species in 25470 trap-nights along the two elevational gradients, with a total successful trapping of 7.88%. The number of individuals captured per transect varied from 92 to 383, and the number of species recorded ranged from 7 to 25 (Supplementary Table S1). Twenty-seven species including 12 insectivores, 16 large-ranged species, and 14 endemic species were captured on the western slope. Thirty-three captured species on the eastern slope included 14 insectivores, 14 large-ranged species, and 19 endemic species. The shared species for the two faces were high, with 23 species occurring on both slopes. The elevational ranges, taxonomy and endemism of all species are shown in Fig. 2.

**Figure 3:**
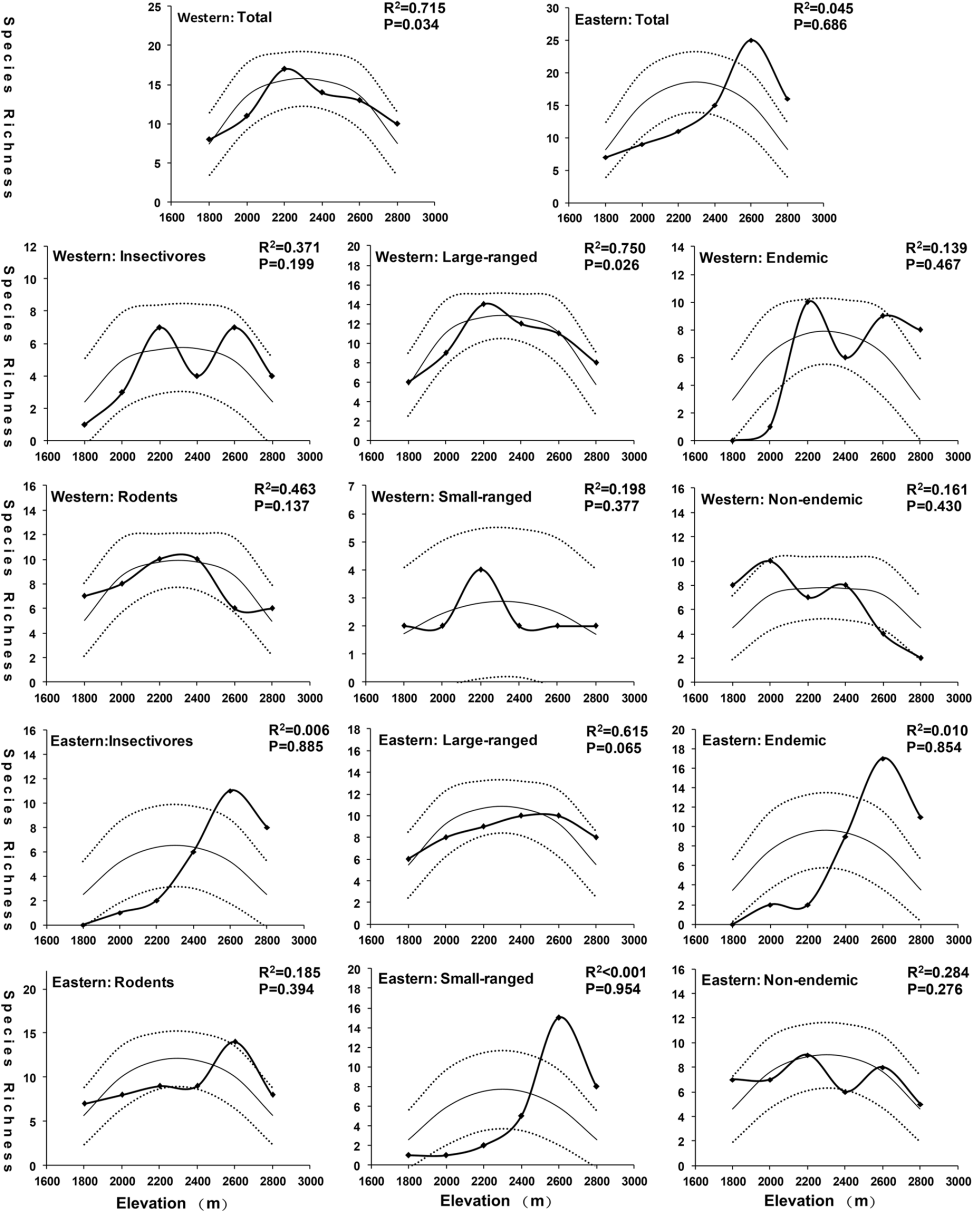
Observed species richness curves of small mammals (line with squares) along the elevation gradient of the Ailao Mountains for both the western and eastern slopes. The MDE-null predicted species richness (lines only) with 95% confidence intervals (dotted lines) is based on 5,000 simulation samples with discrete domain analysis in RangeModel5. The R^2^ and P-values were obtained by linear regressions of the observed richness on the predicted values to estimate the impact of the null model.

Most of the small mammal groups exhibited a hump-shaped relationship with altitude. However, shapes of the curves were varied for the two slopes and among different partitioned groups (Fig. 3). The total species richness on the western slope peaked at mid-elevation (2,200 m), but peaked at a higher elevation (2,600 m) on the eastern slope. The insectivores had two peaks on the western slope, one at 2,200 m and the other at 2,600 m; whereas on the eastern slope, they showed only a single peak at the higher elevation (2,600 m), with species richness decreasing rapidly towards lower elevations. Species richness of rodents peaked at mid-elevations (2,200–2,400 m) on the western slope, but had a higher elevation peak (2,600 m) with a relatively uniform distribution at lower elevations on the eastern slope. The richness pattern of endemic species was similar to that of insectivores, which had two peaks on the western slope (the larger peak at 2,200 m and the smaller peak at 2,600 m) and a single peak at the higher elevation (2,600 m) on the eastern slope. Non-endemic species richness was higher at lower elevations (2,000 m) on the western slope and almost unchanged along the eastern slope. Large-ranged species had peak at 2,600 m on the eastern slope and showed a hump-shaped pattern with a peak at 2,200 m on the western slope. Small-ranged species had a peak at higher elevation (2,600 m) on the eastern slope while on the western slope showed a peak at mid-elevation (2,200 m).

Polynomial regression analyses also revealed that the species richness patterns had some differences for the two slopes (Table 1). Generally, species richness patterns for the western slope were better to fit by a quadratic function with altitude, whereas a linear function was the best fit for the eastern slope.

**Table 1:** Polynomial regressions of different small mammal species richness patterns along elevational gradients in the Ailao Mountains for both the western and eastern slopes.

Ailao Mountain	Total species	Insectivores	Rodents	Large-ranged species	Small-ranged species	Endemic species	Non-endemic species
Western slope							
Linear (R^2^)	0.05	**0.30**	0.10	0.07	**0.02**	0.58*	0.73*
AICc	36.56	**30.98**	29.57	35.29	**20.39**	**35.09**	28.16
Quadratic (R^2^)	**0.84**	0.67	**0.70**	0.89*	0.25	0.73	0.88*
AICc	**31.03**	31.52	**28.04**	**27.28**	23.81	37.38	**28.04**
Cubic (R^2^)	0.84	0.67	0.72	0.89	0.35	0.73	0.89
AICc	40.77	41.52	37.49	37.16	32.89	47.38	37.6
Eastern slope							
Linear (R^2^)	**0.64**	0.82*	**0.25**	0.36	**0.61**	0.75*	**0.14**
AICc	**39.13**	**30.31**	**32.16**	25.26	**37.63**	**37.35**	**26.17**
Quadratic (R^2^)	0.67	0.82	0.38	0.96**	0.61	0.75	0.41
AICc	43.6	35.25	35.94	**14.29**	42.63	42.27	28.92
Cubic (R^2^)	0.86	0.95	0.63	0.99*	0.82	0.91	0.42
AICc	48.65	38.12	42.86	17.23	47.97	46.46	38.78

Bold letters indicate the parameters for each model with the lowest corrected Akaike information criterion (AICc) values.

^*^P < 0.05; **P < 0.01.

### The mid-domain effect (MDE)

The predictive ability of the mid-domain effect (MDE) varied by slope, and among different subsets of the species groups (Fig. 3). For the total number of species, elevational richness pattern on the western slope supported the MDE predictions (R^2^ = 0.751, P = 0.034), whereas MDE contributed little to the pattern on the eastern slope (R^2^ = 0.045, P = 0.686). On both the slopes, large-ranged species fit the MDE null model predictions better than did species with smaller-range sizes. However, there were consistent trends neither in deviations among insectivores and rodents, nor among endemic and non-endemic species.

### Multiple regression accounting for species richness pattern

Bivariate Pearson correlation revealed that MAT and MAH were highly correlated with NDVI (Table 2), hence, only NDVI was used as a surrogate in the multiple regressions. Results of model selection showed that the interactions between slope and other variables are not included in the best models for all the species groups (Table 3), suggesting no significant difference in relationship between small mammal richness and explanatory factors by the slopes. However, the best models varied among different small mammal groups. Area and NDVI were positively correlated with total, insectivores and endemic species richness. They together explained 79.2%, 79.9% and 82.4% of the variance in richness for total, insectivores and endemic species, respectively. MDE and area were positively correlated with rodent richness, and together they accounted for 64.5% of the variance in richness. MDE was the only factor entering in the best model for large-ranged species, and accounted for 79.0% of the variation in richness. The area was the only factor strongly correlated with small-ranged species, and explained 73.7% of the variation in richness. PSR was the only variable having a significant effect on richness pattern of non-endemic species, and accounted for 48.2% of the variance in richness.

**Table 2:** Pearson correlation coefficients between potential drivers in Ailao Mountains.

	MDE	AREA	MAT	MAH	NDVI	PSR
MDE	1.00					
AREA	−0.01	1.00				
MAT	−0.13	−0.40	1.00			
MAH	0.27	0.31	−0.84^**^	1.00		
NDVI	0.52	0.34	−0.87^**^	0.84^**^	1.00	
PSR	0.16	−0.38	0.85^**^	−0.50	−0.61^*^	1.00

*P < 0.05; **P < 0.01.

MDE, the mid-domain effect; MAT, mean annual temperature; MAH, mean annual humidity; NDVI, normalized difference vegetation index; PSR, plant species richness.

**Table 3:** Results of model selection (best model) and model averaging for the richness of different small mammal species groups along elevational gradients in Ailao Mountains.

	Total species	Insectivores	Rodents	Large-ranged species	Small-ranged species	Endemic species	Non-endemic species
**Best model**							
MDE			2.78*	3.13**			
AREA	2.17*	1.96**	2.89*		5.16***	1.95′	
NDVI	2.31**	3.32***				4.87***	
PSR							2.09*
Slope							
Slope:MDE							
Slope:AREA							
Slope:NDVI							
Slope:PSR							
R^2^	0.792	0.797	0.645	0.790	0.737	0.824	0.482
AICc	65.27	50.92	53.18	58.10	49.39	59.63	54.59
**Model averaging**							
MDE	0.26	0.12	0.71	0.75	0.29	0.15	0.24
AREA	0.69	0.43	0.77	0.11	1.00	0.47	0.23
NDVI	0.68	1.00	0.07	0.40	0.24	1.00	0.18
PSR	0.12	0.10	0.04	0.25	0.08	0.19	0.64
Slope	0.13	0.15	0.07	0.11	0.14	0.28	0.15
Slope:MDE							
Slope:AREA					0.02	0.07	
Slope:NDVI	0.01	0.03				0.01	
Slope:PSR		0.01					0.01

The best model was selected based on the lowest corrected Akaike information criterion (AICc) values.

′P < 0.1; *P < 0.05; **P < 0.01; ***P < 0.001.

MDE, the mid-domain effect; MAT, mean annual temperature; MAH, mean annual humidity; NDVI, normalized difference vegetation index; PSR, plant species richness. “:” indicates the interactions between variables.

In the comparison of values of the relative importance of each variable, the model averaging gave similar results as the model selection (Table 3). The interactions between the slope and other factors were also not supported as the main driver for all small mammal groups. For the total species, area and NDVI were also proved to be the two most important factors for richness, however, their roles were slightly changed. Area had higher importance than NDVI in the model averaging, but differed in the model selection. Because of the uncertainty of the best model (i.e. the delta AICc of the top four ranked models only 2.02; Supplementary Table S2), the result of model averaging might be more reliable. NDVI was also approved to be the strongest driver for the richness of insectivores and endemic species, followed by the area. MDE and area were the two most significant factors for rodent richness, with almost the same strength. MDE, area and PSR were ranked as the primary drivers of the richness for large-ranged, small-ranged and non-endemic species, respectively.

## Discussion

Based on data from standardized field surveys, the present study supported the hump-shaped pattern of the total species richness of small mammals along elevational gradients on both the eastern and western slopes of the Ailao Mountains (Fig. 3). Our findings were consistent with most of the elevational gradient studies of small mammals^8,9,17^. However, the elevational diversity curves appeared highly different for the two slopes, with a mid-elevation peak (2,200 m) on the western slope and a higher elevational peak (2,600 m) on the eastern slope. Such a result was not surprising in consideration of the special geography of Ailao Mountains, where both spatial and climatic conditions are quite different between the slopes^29^ (Supplementary Fig. S1).

Our results also revealed elevational diversity curves with different shapes when we partitioned species richness among insectivores and rodents, large-ranged and small-ranged species, and endemic and non-endemic species (Fig. 3). The results supported previous studies that advocated variation in richness patterns in accordance with taxonomy, range size, and endemism^23,26,27^. Compared with small-ranged species, large-ranged species were more likely to show mid-domain peaks in richness patterns. The peaks of endemic species occurred at the higher elevation relative to non-endemic species. Species richness of insectivores decreased more sharply at the low elevation relative to rodents. These findings may indicate that the total species richness pattern could be influenced by species composition in this region.

MDE have been widely discussed as a powerful factor to explain species richness patterns^23,27^. However, the predictive power of MDE for small mammals is inconsistent across different elevational gradients^30^. In some cases, the species richness of small mammals fits well with MDE predictions^17,21^; yet in other cases, its predictive power is very weak^7,11^. The findings of this study had mixed support for MDE on the two slopes of Ailao Mountains. Total species richness of non-flying small mammals fit well with MDE prediction on the western slope, but it showed no significant relationship with MDE on the eastern slope (Fig. 3). McCain^31^ concluded that MDE can be eliminated as the main driver in shaping the elevational diversity of small mammals worldwide, because of the highly variable fits. Our results might support McCain’s conclusion to some extent, because we found high variability in the fit of MDE for the two slopes. Additionally, MDE was not supported as the main driver for both model selection and model averaging (Table 3).

Studies have indicated that the predictive power of MDE also varies among different species groups, with large-ranged and endemic species fitting MDE better than small-ranged and non-endemic species, respectively^17,23^. Our study presents similar results for large-ranged and small-ranged species on both the slopes (Fig. 3). Compared with non-endemic species, endemic species richness peaked at higher elevations, but the fit of MDE did not show a general preference for the endemic and non-endemic species. Further, support of MDE predictions was little better for rodents than insectivores, but for both group it was not statistically significant. Overall, our results highlight the variability in the fit of MDE for the two slopes, and question whether MDE alone can explain the elevational species richness pattern for small mammals in general.

Our results revealed that a combination of area and productivity (i.e. NDVI) can explain most of the variations for total small mammal species richness (Table 3). The results were reasonably consistent with previous studies that have demonstrated both area and productivity as good drivers for biodiversity patterns^18,25^. In our study, since MAT and MAH were highly correlated with NDVI (Table 2), it is difficult to discriminate the relative contribution of these factors. However, we found that the total species richness is positively correlated with the abundance (i.e. individuals; r = 0.755, P = 0.005), which may suggest the important role of productivity in shaping the species richness patterns^32^. However, we found the variance in primary factor for richness pattern among different partitioned groups (Table 3). In contrast to large-ranged species richness, which was strongly correlated with MDE, small-ranged species richness was mostly affected by the area. It confirmed that larger ranged species fit MDE predictions better than species with smaller ranges^27^. Small-ranged species have limited elevational distribution ranges, so it is expected to be highly influenced by the area. Endemic species richness was affected by NDVI and area, while non-endemic species richness was mostly impacted by PSR. This result support the previous studies that endemic species richness is more affected by the area^1,33^, and perhaps more constrained by the local conditions. Non-endemic species are related to PSR as the higher PSR may supply resources for these newly occupied mammals with the lesser competitive ability^33^. The different mechanisms for endemic and non-endemic species could also correlate with their unknown dispersal and evolutionary history^34^. The insectivore species, maybe because of their high endemism (Fig. 2), were strongly affected by NDVI as endemic species. Probability due to the mean elevational range for rodents was significantly larger than insectivores (P < 0.001) in our study, rodents showed a stronger correlation with MDE. The results presented here suggest for the strong influence of species pool in the mechanisms for richness patterns. An improved species-specific knowledge of small mammals is appropriate to improve the understanding of their diversity patterns^28^.

One of our important findings revealed the general underlying mechanisms for each species group, even though species richness patterns differed between the slopes (Fig. 3 and Table 3). Previous studies focused on a single montane gradient and found capricious variance in the explanatory factors for elevational species richness patterns. These differences may be because of the different spatial factors, climate and evolutionary histories of a region among the studies^28^, and also could be strongly influenced by differences in sampling methods employed and a lack of robust explanatory data^14^. In our study, we used standard sampling regime to survey small mammals along gradients in the same mountains, and collected accurate potential factors in shaping the elevational species richness patterns. The small mammal community similarity was high (Sorensen’s similarity index = 76.7) between the slopes, so that species of the two gradients may share similar evolutionary histories. Our results showed that the interactions between the slope and other factors were not significant for all small mammal groups (Table 3). These results indicated that the variation in mechanisms underlying the elevational diversity patterns may not be due to the differences in spatial and climatic conditions. Overall, the results suggested for the existence of a general mechanism driving elevational diversity patterns for a species group within the same region.

## Conclusion

Our results supported a hump-shaped species richness pattern of small mammals along two contrasting elevational gradients in the Ailao Mountains of southwestern China. However, shapes of species richness curves varied for the western and eastern slopes and among differently partitioned species groups (i.e. insectivores vs. rodents, large-ranged vs. small-ranged species, endemic vs. non-endemic species). Area and productivity (highly correlated with MAT and MAH) were documented to be the most important variables explaining the total species richness patterns of small mammals. The primary driver(s) differed among the different subsets of species groups. However, the underlying mechanisms are general for each species group between the two slopes. We suggest that the general mechanism exists for a species group along elevational gradient within a montane region. Broader comparative studies are needed to examine whether the results presented here are common in other taxa, regions and scales.

## Methods

### Ethics approval

The required permissions were obtained from the Department of Forestry of Yunnan Province, China. All methods were performed in accordance with the guidelines and regulations approved by the internal review board of Kunming Institute of Zoology, Chinese Academy of Sciences (approval ID: SMKX-2012023).

### Study area

The present study was conducted in the Ailao Mountains (23°36′-24°44′ N, 100°54′-101°30′ E), Yunnan Province, southwestern China. The Ailao Mountains are the natural transition from southern-subtropical to mid-subtropical climatic zones^29^, and are situated at the junction of the Yunnan-Guizhou Plateau and the Hengduan Mountains. The Ailao Mountains range runs north to south, and is a part of the Indo-Burma biodiversity hotspot. The higher elevation of the eastern slope of Ailao Mountains is the eroded remains of plateau, so the area of the altitudinal bands is much larger for the eastern slope than western slopes (Fig. 1). Climatically, the Ailao Mountains receive summer monsoons from the Pacific and Indian Oceans, and are influenced by cold air from the north during the winter. This creates distinct dry (November to April) and rainy (May to October) seasons. The climates of the western and eastern slopes are quite different (e.g. the eastern slope receives less precipitation and has lower winter temperatures^29^).

The Ailaoshan National Nature Reserve, established in 1981, covers only the higher elevations (most above 2,000 m) of the mountain range. On both slopes, the forest areas above 1800 m are well protected but below it natural forests have been largely replaced by farmland and villages. Sampling in an area with habitat disturbance or fragmentation could create erroneous elevational diversity patterns^35^. Therefore, we conducted our study from the forest boundary at about 1,800 m to the local peak of the mountain range at about 2,850 m.

Different elevational distribution patterns of plant communities on either slope have been well documented^29,36^. Above 1,800 m on the western slope, there are three major vegetation zones: monsoon evergreen broad-leaf forest and pine (*Pinus kesiya*) forest (1,800–2,200 m), moist evergreen broad-leaf forest (2,200–2,800 m) and mossy dwarf forest (above 2,800 m). On the eastern slope, semi-moist evergreen broad-leaf forest and pine (*Pinus yunnanensis*) forest occur between 1,800 and 2,400 m, transitioning to moist evergreen broad-leaf forest from 2,400 to 2,800 m, and to the mossy dwarf forest above 2,800 m.

### Sampling

Small mammals were sampled using standardized techniques along elevational gradients on both the western and eastern slopes. Transect lines were laid out along elevational gradients at intervals of 200 m, and trap stations were limited within 50 m (i.e. 25 m below or above each transect). At elevations below 1,800 m, there was no intact forest for sampling; therefore, six elevational transects were designed on each slope: 1,800 m (1,775–1,825 m), 2,000 m (1,975–2,025 m), 2,200 m (2,175–2,225 m), 2,400 m (2,375–2,425 m), 2,600 m (2,575–2,625 m), and 2,800 m (2,775–2,825 m). Each trap line was georeferenced by two hand-held GPS units (VISTA HCX, GARMIN, Taiwan).

To ensure adequate sampling, we trapped small mammals twice along each transect line, once finishing just before the rainy season (i.e. from March to May 2013), and the next starting just after the rainy season (i.e. from November 2013 to January 2014). During each trapping phase, we set six trap lines along each transect, and 25 to 30 trap stations were spaced approximately 10 m apart along each trap line. A Sherman trap and a snap trap were set paired on the ground at each station 1–2 m apart. In addition, five pitfalls (plastic buckets that were 14 cm in diameter and 20 cm in depth) were set for shrews around the trap stations in each trap line. The traps were set for 3 consecutive nights at each station. As a result, we standardized the trapping effort at approximately 1,100 trap nights per season at each transect gradient. All small mammals observed on the gradient for the first time were recorded. All captured individuals were identified to species level, weighed and measured. Voucher specimens for each species were prepared as stuffed skins and cleaned skulls. All specimens are stored at Kunming Institute of Zoology, Chinese Academy of Sciences.

### Area data

The area at each elevational band was calculated from digital elevation models (DEM) with a horizontal grid spacing of 30 m resolution downloaded from the Geospatial Data Cloud site, Computer Network Information Center, Chinese Academy of Sciences (http://www.jspacesystems.or.jp/ersdac/GDEM/E/1.html). In order to obtain more accurate data, a five kilometer long plot (nearly covering the entire sampling area) was extracted from each sample transect. We calculated planimetric and surface areas in ArcGIS 10.3 and ENVI 5.2. Because surface area and planimetric area were highly correlated, we used planimetric area in the analyses to facilitate the comparison with previous studies.

### Climate variables

We measured mean annual temperature (MAT) and mean annual humidity (MAH) at each elevational band using temperature and humidity data loggers (DS1923, Thermochron® iButton®, San Jose Semiconductor/Maxim, CA, USA). Data loggers were installed under the forest canopy in the center of the elevational band. The data loggers were set to record temperature and humidity every two hours for one year (from June 1^st^, 2013 to May 31^st^, 2014). We checked the loggers and downloaded data on every six months. Two data loggers (one located at 1,800 m on the western slope and the other at 2,000 m on the eastern slope) were damaged in the wet season. Hence, we estimated these missing data from dry season records and their positional relationships with neighboring elevational data.

### Productivity and vegetation variables

The normalized difference vegetation index (NDVI) was used as an indicator of productivity, which can drive species richness patterns along elevational gradients^11,26^. The NDVI was calculated at each elevational band using the Landsat-8 OLI image dataset downloaded from the United States Geological Survey (USGS). Similar to the calculation of area, five kilometers of each sampling transect were extracted using ENVI 5.2, and NDVI was calculated using the formula [NDVI = (ρ_nir_ − ρ_red_)/(ρ_nir + _ρ_red_)], where ρ_nir =_ near infrared band and ρ_red = _visible red band. The plant species richness (PSR) data were obtained from Niu, *et al*.^36^. If no vegetation data were available for a given transect, the plant species richness was estimated using a linear model based on nearby site records.

### Data analyses

The observed richness and interpolated richness were used to estimate the total number of small mammal species. Observed richness was defined as the total number of species detected (including both trapped and observed) in each sampling transect. Interpolated richness assumes a species is present at a given elevation if it is detected at both higher and lower elevations^8,18,21^. Because of the patterns of the interpolation and observed richness were quite similar (Supplementary Fig. S1) and strongly correlated (western slope: r = 0.978, eastern slope: r = 0.984; P < 0.001), we only used the observed richness for further analyses.

Observed small mammal species were grouped into insectivores and rodents, large-ranged and small-ranged species, and endemic and non-endemic species. Large-ranged species were defined as species with elevational distribution ranges larger than the median size. Endemic species referred to those only occurring in the Hengduan Mountains and the Eastern-Himalaya region. In consideration of its phylogeny (Superorder: Euarchontoglires) and habits, *Tupaia belangeri* was assigned to rodents. We used polynomial regression to estimate the distribution patterns of each of the species groups mentioned above.

RangeModel 5.0^37^ was used to analyze the impact of MDE on species richness. As our sampling was evenly spaced and discrete, we applied discrete domain analysis^37^. The expected mean richness and its 95% confidence interval (CI) were calculated based on 5,000 simulations sampled without replacement. We calculated MDE predictions for all species groups mentioned above. Linear regression was performed between species richness and log-transformed MDE prediction values to estimate the impact of the null model for each species group. In addition, we also used MDE predicted richness as a candidate predictor in the multiple regression models^16^.

The elevational variables (area, MAT, MAH, NDVI and PSR; Figure S2) were log-transformed to obtain the normality and homoscedasticity of the data. We performed generalized linear model (GLM) to explore the underlying mechanisms of each small mammal group. Bivariate Pearson correlation was applied to test the relationship between the variables. All but one of highly correlated factors (|r| > 0.7) was dropped and only one of them was used as a surrogate in the following analyses to confront multicollinearity^38^. Because MAT and MAH were found highly correlated with NDVI (r > 0.84; Table 2), productivity may reflect the optimal combination of temperature and water availability, where the sites with warm and humid climate have higher productivity^4,26^. To reduce the potential collinearity problems, we therefore dropped MAT and MAH and used only NDVI as a surrogate in the multiple regressions. To assess if the underlying mechanisms of small mammal richness along elevational gradients were different between slopes, we also included slope and its interactions with all other predictors into the GLMs. Therefore, we built our global model as: species richness = slope*(MDE + area + NDVI + PSR). We selected the best model based on the lowest corrected Akaike information criterion (AICc) values using package “MuMIn”^39^. We further checked the homoscedasticity of each of the best models using plots of standardized residuals against predicted values. To further account for collinearity, we calculated the variance inflation factor (VIF) of each variable in the best model. Since all the VIFs were <5, collinearity was not a problem in the models^38^. We calculated the variable importance for each factor in the best model with package “caret”^40^. To evaluate the effect of spatial autocorrelation, we calculated Moran’s *I* values for the residuals of each best model in the package “spdep”^41^. Because none of the residuals of the best model was significant (Supplementary Table S3), the influence of spatial autocorrelation should be weak^42^. Further, because of the uncertainty of model-selection, we used model averaging to assess the relative importance of each variable by summing the weights of the models whose cumulative weights equal to 0.95^43^. All the statistical analyses were performed in R 3.3.2 ^44^.

## Electronic supplementary material

Supplementary information
