# Supplementary material for: Patterns and underlying mechanisms of non-volant small mammal richness along two contrasting mountain slopes in southwestern China
Source: Sci Rep. 2017 Oct 16;7:13277. doi: 10.1038/s41598-017-13637-0 (PMC5643442; doi:10.1038/s41598-017-13637-0)

Patterns and underlying mechanisms of non-volant small mammal richness along two contrasting mountain slopes in southwestern China

Zhongzheng Chen, Kai He, Feng Cheng, Laxman Khanal & Xuelong Jiang

Supplementary information

Table S1. Number of individuals, interpolated richness and different species groups of observed richness (total, insectivores, rodents, large-ranged, small-ranged, endemic and non-endemic species) in each elevation on the western and eastern slope of the Ailao Mountains.

				Observed richness						
Slope	Elevation (m)	Individuals	Interpolated richness	total	Insectivores	Rodents	Large-ranged species	Small-ranged species	Endemic species	Non-endemic species
Western	1800	95	8	8	1	7	6	2	0	8
Western	2000	134	11	11	3	8	9	2	1	10
Western	2200	217	20	17	7	10	14	4	10	7
Western	2400	136	18	14	4	10	12	2	6	8
Western	2600	100	15	13	7	6	11	2	9	4
Western	2800	175	10	10	4	6	8	2	8	2
Eastern	1800	108	7	7	0	7	6	1	0	7
Eastern	2000	94	9	9	1	8	8	1	2	7
Eastern	2200	99	13	11	2	9	9	2	2	9
Eastern	2400	92	18	15	6	9	10	5	9	6
Eastern	2600	373	26	25	11	14	10	15	17	8
Eastern	2800	383	16	16	8	8	8	8	11	5
Sum		2006	37	37	1	7	6	2	0	8

Table S2. The top four ranked models of the model selection for the total species pattern in Ailao Mountains.

Model	AREA	MDE	NDVI	AICc	ΔAICc
1	0.65		6.13	65.27	0
2			7.81	66.16	0.89
3	0.97	1.21		66.57	1.30
4	0.91			67.30	2.02

Table S3. Moran’s *I* and P values for residuals of the best models for the richness of different small mammal species groups along elevational gradients in Ailao Mountains.

	Total species	Insectivores	Rodents	Large-ranged species	Small-ranged species	Endemic species	Non-endemic species
Moran’s *I*	-0.01	-0.08	-0.21	0.07	-0.12	-0.33	0.05
P	0.31	0.48	0.75	0.19	0.57	0.91	0.21

Figure S1. Comparison of the observed and interpolated total species richness for non-flying small mammal patterns along elevational gradients in the Ailao Mountains for (a) western slope and (b) eastern slope.

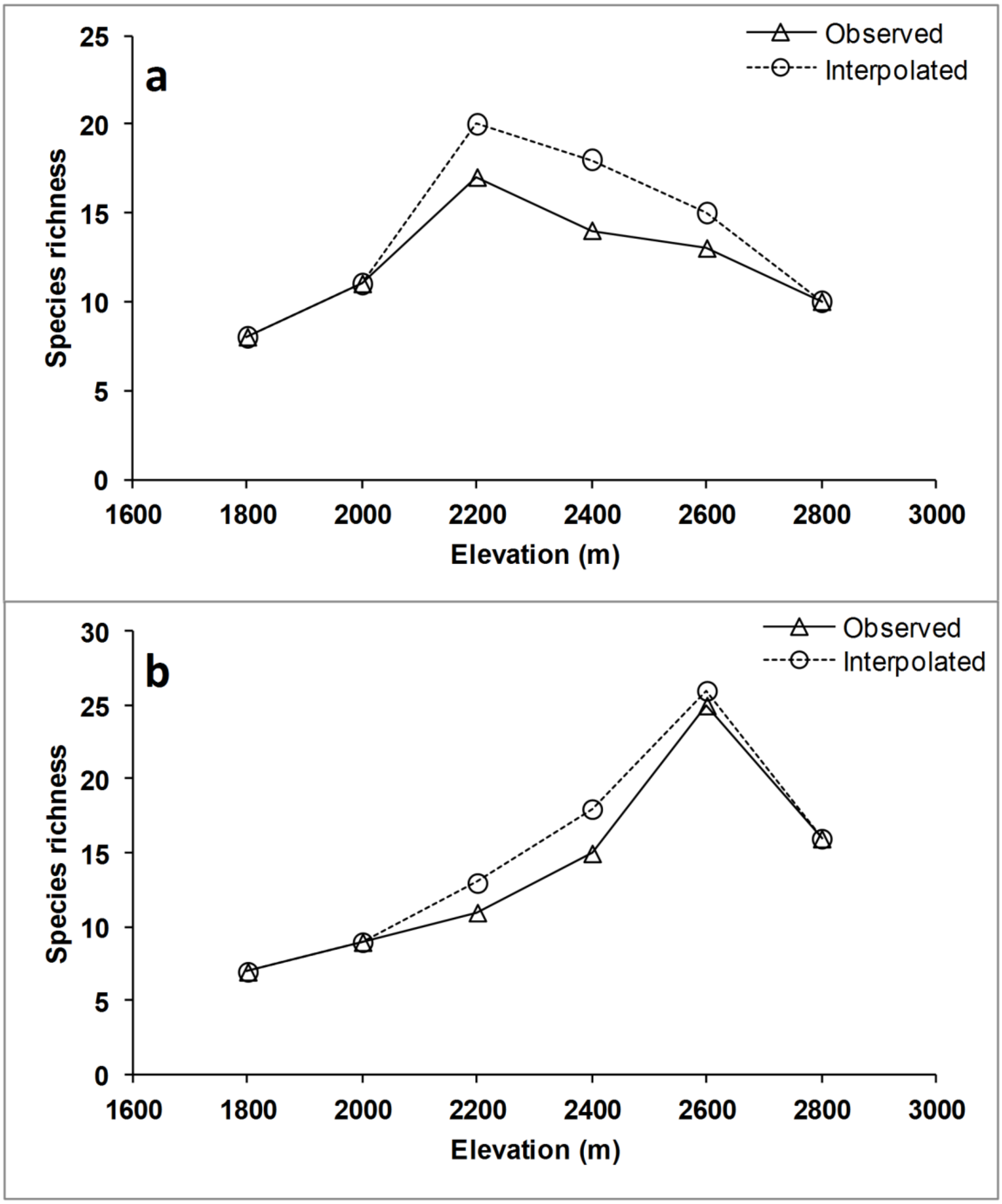

Figure S2. Elevational distribution in the Ailao Mountains for both the western (dotted lines with triangle) and eastern slopes (lines with squares) for (a) area, (b) mean annual temperature (MAT), (c) mean annual humidity (MAH), (d) normalized difference vegetation index (NDVI) and (e) plant species richness (PSR).

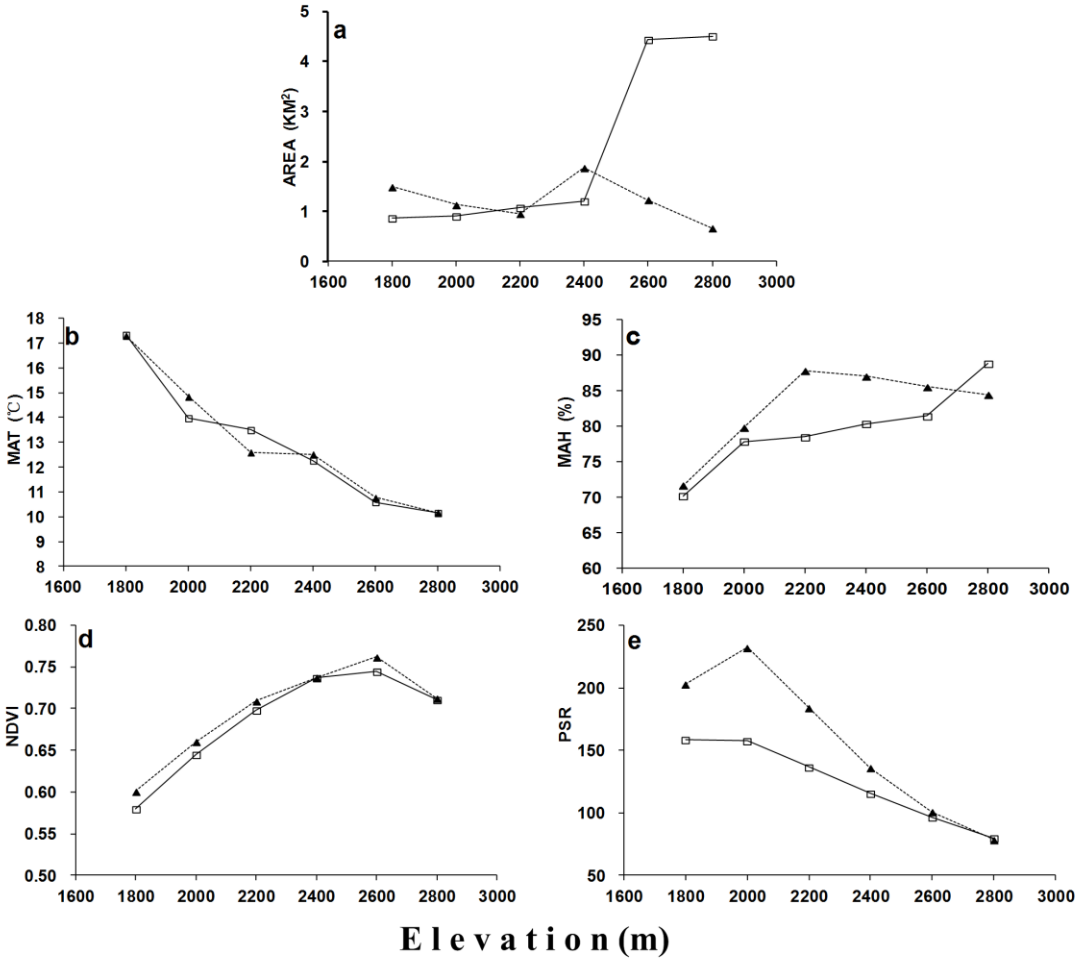

Supplement: Supplementary file 1 — Supplementary information [file 41598_2017_13637_MOESM1_ESM.pdf]
